# Pleiotropic Quantitative Trait Loci (QTL) Mining for Regulating Wheat Processing Quality- and Yield-Related Traits

**DOI:** 10.3390/plants13182545

**Published:** 2024-09-10

**Authors:** Jie Zhao, Lijing Sun, Mengyun Hu, Qian Liu, Junjie Xu, Liming Mu, Jianbing Wang, Jing Yang, Peinan Wang, Qianying Li, Hui Li, Yingjun Zhang

**Affiliations:** 1 Hebei Key Laboratory of Crop Genetics and Breeding, Institute of Cereal and Oil Crops, Hebei Academy of Agriculture and Forestry Sciences, Shijiazhuang 050035, China; zhaojierice@163.com (J.Z.); sunlijing2010@163.com (L.S.); ziren80@163.com (M.H.); liuqian_mbb@126.com (Q.L.); lqianying2022@163.com (Q.L.); zwslihui@163.com (H.L.); 2 Dingxi Academy of Agricultural Sciences, Dingxi 743000, China; muliming2790@163.com (L.M.);

**Keywords:** wheat, GWAS, processing quality, grain yield, pleiotropic QTL

## Abstract

To investigate the genetic basis of processing quality- and yield-related traits in bread wheat (*Triticum aestivum* L., AABBDD), a systematic analysis of wheat processing quality- and yield-related traits based on genome-wide association studies (GWASs) of 285 regional test lines of wheat from Hebei province, China, was conducted. A total of 87 quantitative trait loci (QTL), including twenty-one for water absorption (WA), four for wet gluten content, eight for grain protein content, seventeen for dough stability time (DST), thirteen for extension area (EA), twelve for maximum resistance (MR), five for thousand-grain weight (TGW), one for grain length, and six for grain width were identified. These QTL harbored 188 significant single-nucleotide polymorphisms (SNPs). Twenty-five SNPs were simultaneously associated with multiple traits. Notably, the SNP *AX-111015470* on chromosome 1A was associated with DST, EA, and MR. SNPs *AX-111917292* and *AX-109124553* on chromosome 5D were associated with wheat WA and TGW. Most processing quality-related QTL and seven grain yield-related QTL identified in this study were newly discovered. Among the surveyed accessions, 18 rare superior alleles were identified. This study identified significant QTL associated with quality-related and yield-related traits in wheat, and some of them showed pleiotropic effects. This study will facilitate molecular designs that seek to achieve synergistic improvements of wheat quality and yield.

## 1. Introduction

Bread wheat (*Triticum aestivum* L., AABBDD) feeds over 35% of the world’s population and is a crucial source of carbohydrates, proteins, minerals, and vitamins for humans [1]. Thus, improvements in wheat yield and quality are key considerations for wheat breeders. The main yield-related traits of wheat grain are thousand-grain weight (TGW), grain length (GL), and grain width (GW); the end-use quality of wheat is mainly determined by protein- and starch-related traits, such as grain protein content (GPC) and dough rheological properties. GPC is a primary determinant of wheat nutritional value and baking quality [2]; therefore, it is a key marketing characteristic. Gluten, a complex mixture of polymeric glutenins and monomeric gliadins, confers viscosity and elasticity to dough; accordingly, it is the most important wheat protein. The rheological property of dough, an indicator of its processing quality for noodles, steamed buns, breads, cakes, and other final products [3], can be assessed using farinographic and extensographic parameters [1]. Farinographic parameters include water absorption (WA), dough development time (DDT), and dough stability time (DST), which are used to assess kneading characteristics [4]. Extensographic parameters include the extension area (EA) and maximum resistance (MR), which comprise indicators of dough quality during proofing.

The underlying genetic architecture of grain yield [2,5,6,7,8] and the end-use quality traits [1,4,9,10,11,12] of wheat have been evaluated in multiple quantitative trait loci (QTL) mapping studies. Both relatively high-frequency QTL and significant QTL clusters have been identified. For example, eight stable loci on chromosomes 2A, 2D, 3B, 4A, 5B, 5D, and 7D were mapped in wheat grown under different environmental conditions, revealing associations with wheat yield [13]. Significant QTL for GPC and wet gluten content (WGC), explaining 53.04% and 36% of the phenotypic variation, respectively, were identified on chromosome 5A [14]; chromosomes 3B and 4D each harbored one QTL cluster (for milling quality and starch functionality, respectively) [15]. Major QTL for starch content, WGC, and the Zeleny sedimentation value were detected on chromosome 5D [16]. Lou identified 64 stable quality-related QTL, including some with pleiotropic effects, on chromosomes 6A and 5D [11].

Rapid developments in sequencing technology and high-density marker genotyping have enhanced the use of the genome-wide association study (GWAS) in efforts to determine the genetic basis of quantitative variation in complex traits among species such as wheat [17,18]. A GWAS is more efficient than traditional biparental linkage mapping analyses in terms of analyzing complex traits; the detected markers have a higher degree of polymorphism, more uniform distribution, and greater repeatability. In wheat, GWAS has been used to detect quality-associated loci, including GPC [19,20,21], WA [14,20,22], WGC [11], DDT [22], and DST [22]. A GWAS of 372 diverse European wheat varieties identified two major loci for GPC [21]. Using a GWAS approach, Guo identified 29 unconditional QTL and 13 conditional QTL for GPC, glutenin macropolymer content, amylopectin content, and amylose content during wheat grain development [23]. Similarly, Yang identified 40 core quantitative trait nucleotide (QTN) regions for six grain quality traits and three dough rheological properties [12]. However, considering the complexity of detecting wheat quality-related traits and the effects of genetic background on wheat populations, the contributions of most previously identified QTL were small and their phenotypic accuracies were low, thus limiting the application of quality-related QTL in wheat breeding [24]. Additionally, neither the extenso-graph parameters for dough nor the genetic basis of the synergistic regulation of wheat grain yield and quality have been adequately investigated.

In this study, a QTL analysis using the wheat 15K SNP array was performed for six quality-related traits (WA, WGC, GPC, DST, EA, and MR) and three grain yield-related traits (TGW, GL, GW) across 285 regional test lines of wheat in China. We aimed to use the GWAS approach to identify QTL for quality- and yield-related traits, thereby identifying important QTL for use in wheat breeding programs. A second QTL analysis was performed for wheat quality and yield traits such as GPC, dough rheological properties, and grain weight and shape; we sought to determine their relationships at the QTL level and detect trade-offs between grain yield and quality. 

## 2. Results

### 2.1. Phenotype Assessments

Significant differences in the nine detected traits were identified among the test lines of wheat. The average WA, WGC, GPC, DST, EA, MR, TGW, GL, and GW in the surveyed accessions were 60.68% (range: 53.60–69.00%), 29.99% (22.50–45.20%), 14.68% (12.20–17.40%), 11.04 min (1.20–47.40 min), 72.34 cm2 (19.00–227.00 cm^2^), 351.10 EU (72.00–989.00 EU), 38.08 g (27.11–50.23 g), 6.35 mm (5.49–7.00 mm), and 3.16 mm (2.74–3.56 mm), respectively. The coefficient of variation ranged from 4.59% to 78.05%, indicating large differences in the detected traits for all genotypes (Table 1).

**Figure 1:**
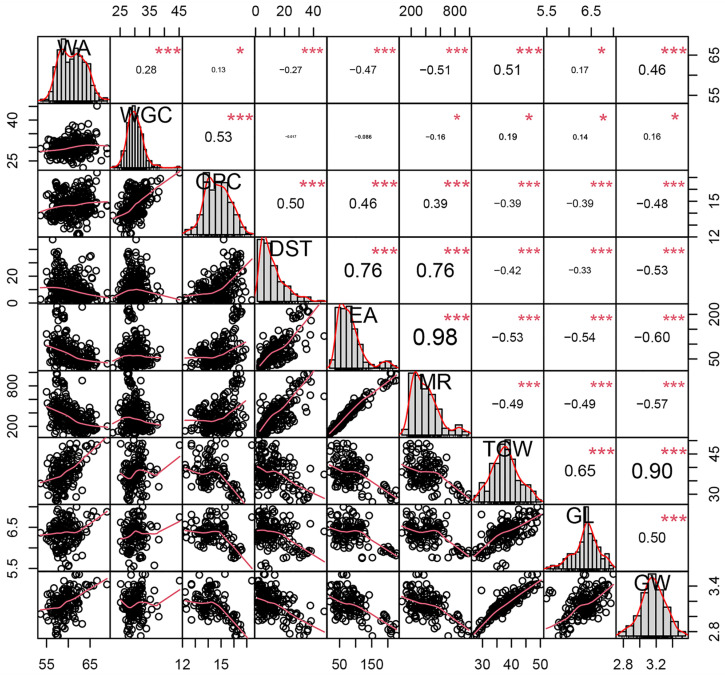
Phenotypic performances, distributions, and correlation coefficients of traits in 285 wheat (*Triticum aestivum* L.) accessions. The frequency distribution of phenotypic data for each trait is shown in histogram format. *X*-*Y* scatter plots include adjusted Pearson’s coefficients. * *p* ≤ 0.05, *** *p* ≤ 0.001. WA, water absorption; WGC, wet gluten content; GPC, grain protein content; DST, dough stability time; EA, extension area; MR, maximum resistance; TGW, thousand-grain weight; GL, grain length; GW, grain width.

In pairwise analyses of correlations between the detected traits (Figure 1), positive correlations were identified in all comparisons of GPC, DST, EA, and MR (GPC vs. DST: *r* = 0.50 ***, GPC vs. EA: *r* = 0.46 ***, GPC vs. MR: *r* = 0.39 ***, DST vs. EA: *r* = 0.76 ***, DST vs. MR: *r* = 0.76 ***, and EA vs. MR: *r* = 0.98 ***). WA was positively correlated with WGC (*r* = 0.28 ***), TGW (*r* = 0.51 ***), and GW (*r* = 0.46 ***). Weakly positive correlations were detected between WA and GPC (*r* = 0.13 *) and between WA and GL (*r* = 0.17 *); WA displayed negative correlations with DST (*r* = −0.27 ***), EA (*r* = −0.47 ***), and MR (*r* = −0.51 ***). WGC was positively correlated with GPC (*r* = 0.53 ***) and weakly positively correlated with TGW (*r* = 0.19 *), GL (*r* = 0.14 *), and GW (*r* = 0.16 *); it showed a weakly negative correlation with MR (*r* = −0.16 *) but no significant correlations with DST or EA. TGW, GL, and GW exhibited negative correlations with GPC, DST, EA, and MR, with *r* values ranging from −0.33 to −0.60. In all pairwise comparisons of TGW, GL, and GW, the correlations were positive (TGW vs. GL: *r* = 0.65 ***, TGW vs. GW: *r* = 0.90 ***, and GW vs. GL: *r* = 0.50 ***).

### 2.2. Linkage Disequilibrium (LD) Analysis and Population Structure

**Figure 2:**
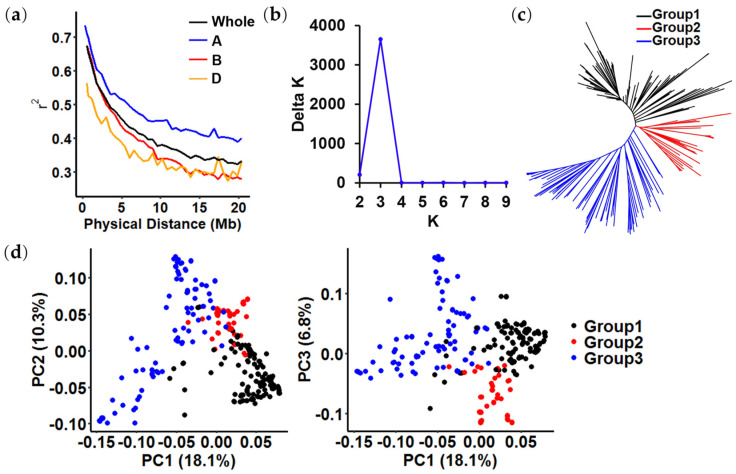
Population structure analysis of the 285 wheat accessions. (**a**) Linkage disequilibrium decay values for the whole genome and the A, B, and D sub-genomes of wheat; (**b**) population structure analysis using Structure software; (**c**) neighbor-joining tree of all wheat accessions; (**d**) principal component analysis showing the population structure in the diversity panel.

The average ranges of LD values for the A, B, and D sub-genomes and the whole genome, defined as the physical distance needed for r^2^ to decay to half of its maximum value, were approximately 20 Mb, 10 Mb, 10 Mb, and 13 Mb, respectively (Figure 2a).

Three methods were used to cluster the accessions and assess similarity, with the following results. First, the delta K value reached a sharp peak at K = 3 in the plot of K vs. ΔK, according to the Structure software (Figure 2b). Second, phylogenetic tree analysis classified the panel into three subpopulations (Figure 2c). Finally, despite sufficient admixture, principal component analysis (PCA) showed that the panel was divided into three major groups (Figure 2d).

### 2.3. GWAS of the Grain Quality-Related Traits

**Figure 3:**
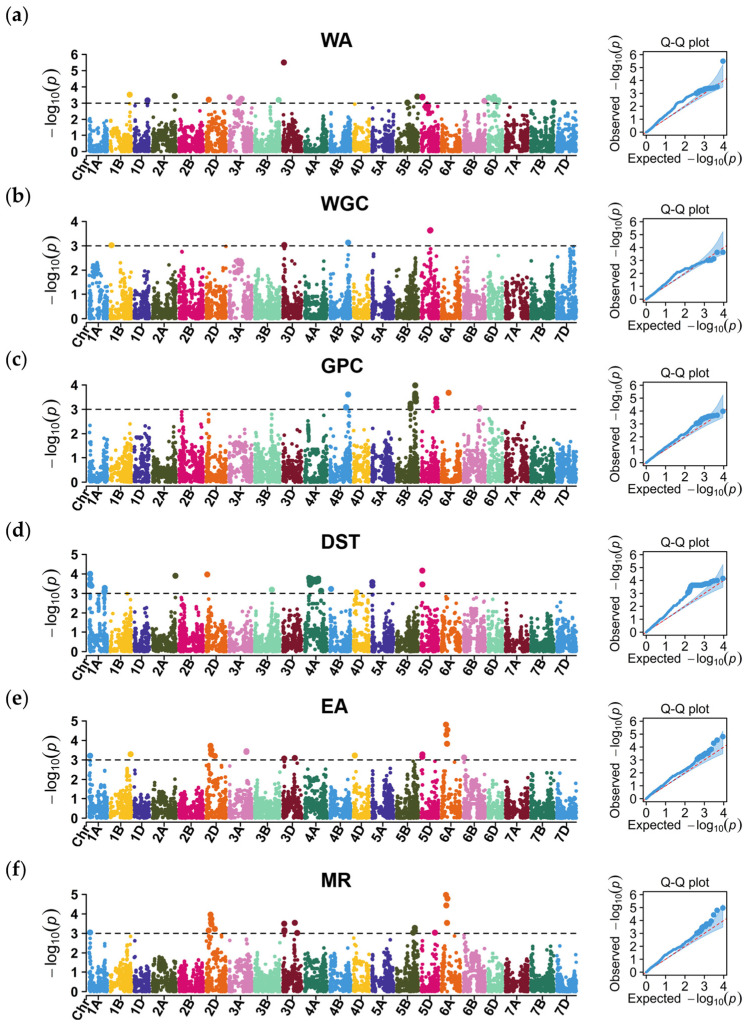
Manhattan and quantile–quantile (Q-Q) plots for (**a**) water absorption (WA), (**b**) wet gluten content (WGC), (**c**) grain protein content (GPC), (**d**) dough stability time (DST), (**e**) extension area (EA), and (**f**) maximum resistance (MR).

The GWAS identified 162 marker–trait associations (MTAs), representing 75 QTL for grain quality traits on almost all wheat chromosomes (except 2B, 7A, and 7D) (Figure 3; Table S1).

#### 2.3.1. Water Absorption

Twenty-one QTL for WA, represented by 24 MTAs, were detected on chromosomes 1B, 1D, 2A, 2D, 3A, 3B, 3D, 5B, 5D, 6B, 6D, and 7B (Figure 3a). Of these, chromosome 3A had six QTL for WA; these were represented by six different MTAs and explained 23.52% of the phenotypic variation. Four QTL were represented by five different MTAs, at 15.57 Mb, 152.26 Mb, 196.57–198.13 Mb, and 333.49 Mb (all on chromosome 6D), and explained 3.93–4.25% of the phenotypic variation. The QTL *qWA-5B.1* and *qWA-5B.2* were mapped to chromosome 5B, explaining 3.72–4.27% of the phenotypic variation. An important QTL, *qWA-3D*, was marked by *AX-108907834*, which was mapped to position 28.13 Mb on chromosome 3D and explained 7.51% of the phenotypic variation. Additionally, eight QTL, distributed on chromosomes 1B, 1D, 2A, 2D, 3B, 5D, 6B, and 7B, explained 3.73–4.45% of the phenotypic variation.

#### 2.3.2. Wet Gluten Content

Four QTL, represented by six MTAs, were detected on chromosomes 1B, 3D, 4B, and 5D (Figure 3b). *qWGC-5D*, represented by two MTAs on chromosome 5D (290.06–302.00 Mb), explained 10.61% of the phenotypic variation for WGC. *qWGC-3D* was represented by two MTAs that mapped to position 42.30–42.41 Mb on chromosome 3D and explained 8.51% of the phenotypic variation. The QTL *qWGC-1B* and *qWGC-4B*, represented by *AX-109435258* (42.30 Mb on chromosome 1B) and *AX-109488694* (593.13 Mb on chromosome 4B), explained 4.26% and 4.44% of the phenotypic variation, respectively.

#### 2.3.3. Grain Protein Content

Twenty-four MTAs were associated with eight loci and explained 3.21–4.45% of the phenotypic variation for GPC (Figure 3c). Two QTL on chromosome 4B explained 16.34% and 3.96% of the phenotypic variation, respectively. These two QTL were represented by five MTAs and one MTA, respectively. Three QTL on chromosome 5B included three (position 455.53–455.97 Mb), eight (605.16–611.93 Mb), and two (634.45–634.99 Mb) MTAs, respectively. Of these, *qGPC-5B.2* was represented by eight single-nucleotide polymorphisms (SNPs) and explained 31.29% of the phenotypic variation. One QTL was represented by three SNPs, detected at 493.45–497.83 Mb on chromosome 5D and explaining 3.30–3.72% of the phenotypic variation. Additionally, two QTL marked by two different MTAs were detected on chromosomes 6A and 6B and explained 3.24% and 4.04% of the phenotypic variation, respectively.

#### 2.3.4. Dough Stability Time

Seventeen QTL associated with DST, represented by 65 MTAs, were detected on chromosomes 1A, 2A, 2D, 3B, 4A, 4B, 4D, 5A, and 5D (Figure 3d). Three QTL (*qDST-1A.1*, *qDST-1A.2*, and *qDST-1A.3*) on chromosome 1A were represented by four, one, and seven MTAs, respectively, explaining 2.81–3.88% of the phenotypic variation. Three QTL (*qDST-2A*, *qDST-2D*, and *qDST-3B*), marked by *AX-109384630*, *AX-94458060*, and *AX-109946806*, respectively, explained 2.97–3.85% of the phenotypic variation. Seven QTL on chromosome 4A explained 2.90–3.64% of the phenotypic variation. These included the QTL *qDST-4A.5*, represented by 15 MTAs at 349.91–423.37 Mb on chromosome 4A, which explained 3.43–3.55% of the phenotypic variation. Three QTL (*qDST-4A.2*, *qDST-4A.3*, and *qDST-4A.6*) were represented by eight, nine, and four MTAs, respectively, explaining 3.25–3.60% of the phenotypic variation. Two QTL (*qDST-4A.4* and *qDST-4A.7*), each represented by three different MTAs (including MTAs at 302.81–310.23 Mb and 540.34–541.33 Mb on chromosome 4A, respectively), explained 2.90–3.53% of the phenotypic variation. Finally, the QTL *qDST-4A.1*, represented by *AX-111164022* (at 149.65 Mb on chromosome 4A), explained 3.64% of the phenotypic variation.

#### 2.3.5. Extension Area

Thirteen QTL for EA, marked by 21 MTAs, were detected on chromosomes 1A, 1B, 2D, 3A, 3D, 4D, 5D, 6A, and 6B (Figure 3e). Among these, nine QTL—*qEA-1A*, *qEA-1B*, *qEA-2D.2*, *qEA-3D.1*, *qEA-3D.2*, *qEA-4D*, *qEA-6A.2*, *qEA-6A.3*, and *qEA-6B*—were represented by the MTAs *AX-111015470*, *AX-109376365*, *AX-110426295*, *AX-110928411*, *AX-110000775*, *AX-109230716*, *AX-109974589*, *AX-110931567*, and *AX-108924932*, respectively, explaining 2.93–4.66% of the phenotypic variation. One significant QTL, *qEA-2D.1*, was represented by six MTAs, which were marked at 143.23–175.28 Mb on chromosome 2D and explained 3.18–3.69% of the phenotypic variation. Furthermore, three QTL (*qEA-3A*, *qEA-5D*, and *qEA-6A.1*) were represented by two MTAs, respectively, explaining 3.32–4.37% of the phenotypic variation.

#### 2.3.6. Maximum Resistance

Twelve QTL, including 22 MTAs, were detected on chromosomes 1A, 2D, 3D, 5B, 5D, and 6A; they explained 3.12–5.63% of the phenotypic variation (Figure 3f). The QTL *qMR-1A* and *qMR-5D*, represented by *AX-111015470* and *AX-111198874*, explained 3.15% and 3.13% of the phenotypic variation, respectively. The QTL *qMR-2D.1*, *qMR-2D.2*, and *qMR-2D.3*, mapped to chromosome 2D, explained 3.27–4.30% of the phenotypic variation. Six MTAs marked *qMR-2D.2* and collectively explained 23.75% of the phenotypic variation. Three QTL on chromosome 3D explained 3.12–3.77% of the phenotypic variation. Additionally, two QTL on chromosome 5B and another two QTL on chromosome 6A explained 3.13–3.44% and 3.76–5.63% of the phenotypic variation, respectively.

### 2.4. GWAS of the Grain Yield-Related Traits

**Figure 4:**
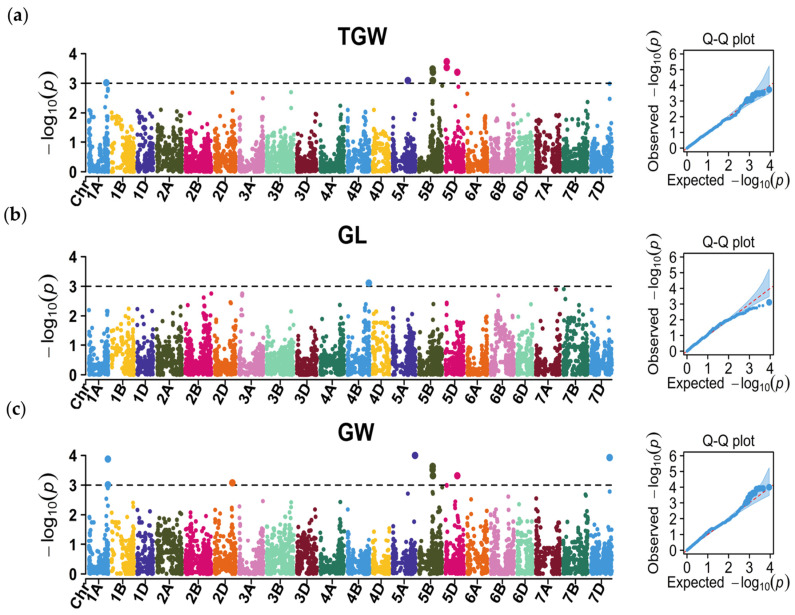
Manhattan and Q-Q plots for (**a**) thousand-grain weight (TGW), (**b**) grain length (GL), and (**c**) grain width (GW).

The GWAS identified 26 MTAs, representing 12 QTL for TGW, GL, and GW, on chromosomes 1A, 2D, 4B, 5A, 5B, 5D, and 7D (Figure 4; Table S2).

Five QTL associated with TGW were identified on chromosomes 1A, 5A, 5B, and 5D. A key QTL, *qTGW-5B*, was composed of seven significant SNPs and explained 11.10% of the total phenotypic variation for TGW. Another QTL for TGW, *qTGW-5D.1*, was represented by two SNPs at positions 38.93–40.34 Mb on chromosome 5D. Additionally, three QTL (*qTGW-1A*, *qTGW-5A*, and *qTGW-5D.2*) for TGW were detected on chromosomes 1A, 5A, and 5D, respectively; they collectively explained 2.76% of the phenotypic variation.

*qGL-4B*, represented by the SNP *AX-111057902*, was located at 652.37 Mb on chromosome 4B and explained up to 5.55% of the phenotypic variation in GL.

GW was associated with six QTL on chromosomes 1A, 2D, 5A, 5B, 5D, and 7D. *qGW-5A*, marked by SNP *AX-95213349* at position 685.43 Mb on chromosome 5A, was the most significant QTL, explaining 8.13% of the phenotypic variation. Another three QTL (*qGW-1A*, *qGW-2D*, and *qGW-7D*), on chromosomes 1A, 2D, and 7D, were represented by two SNPs, respectively; they explained 5.76–7.95% of the phenotypic variation for GW. Notably, the QTL *qGW-5B* (on chromosome 5B) and *qGW-5D* (on chromosome 5D) had a position similar or identical to *qTGW-5B* and *qTGW-5D.2*, respectively.

### 2.5. Analysis of Pleiotropic and Flour Quality-Related and Grain Yield-Related MTAs

Many MTAs were simultaneously associated with multiple traits. For example, *AX-109488694*, at 593.13 Mb on chromosome 4B, was associated with WGC and GPC. *AX-111015470*, at 29.78 Mb on chromosome 1A, was associated with DST, EA, and MR. Two MTAs (*AX-110978247* and *AX-89603436*), at 34.79 Mb and 37.13 Mb on chromosome 5D, respectively, were associated with both DST and EA. Additionally, 13 MTAs for EA, including all MTAs detected on chromosomes 2D, 3D, and 6A, were simultaneously associated with DST. TGW had six SNPs (*AX-108958865*, *AX-110576628*, *AX-108827033*, *AX-111672485*, *AX-111030246*, and *AX-108735670*) in common with GW. Both *AX-111917292* and *AX-109124553*, at 38.93 Mb and 40.34 Mb on chromosome 5D, were associated with WA and TGW.

### 2.6. Effects of Superior Alleles on Phenotypes and Allelic SNPs on Wheat Quality and Yield

**Figure 5:**
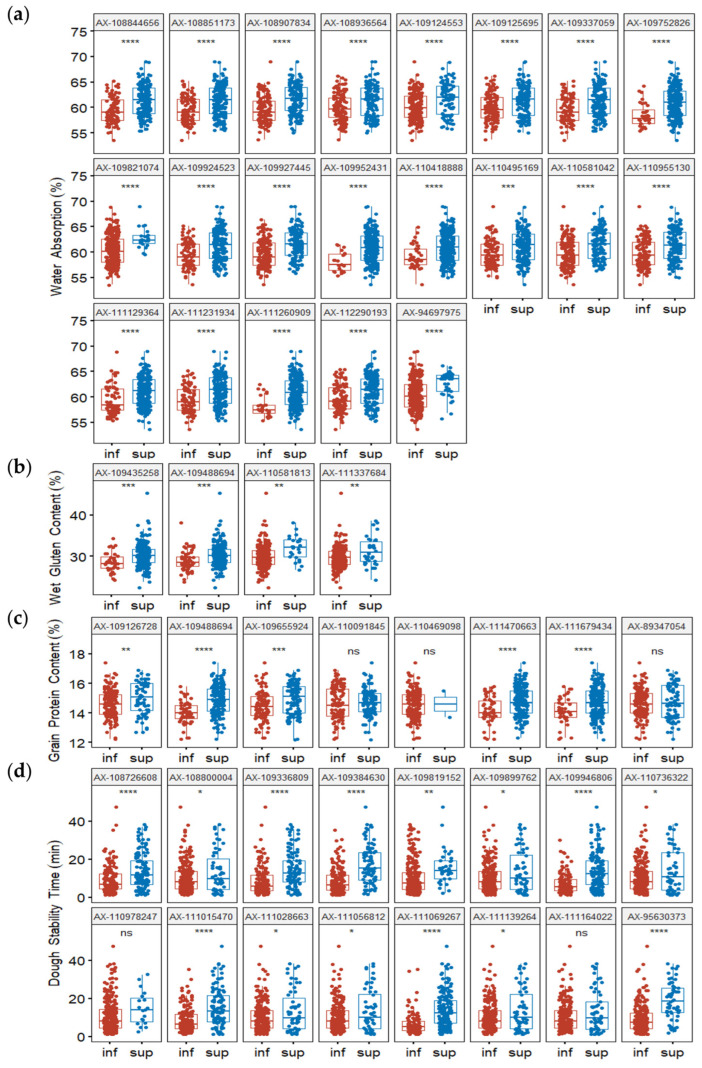
Average phenotypic values of accessions with different alleles for water absorption (**a**), wet gluten content (**b**), grain protein content (**c**), and dough stability time (**d**). *p*-values were determined by two-tailed Student’s *t*-tests. ns: no significant difference, *p* > 0.05; * *p* < 0.05; ** *p* < 0.01; *** *p* < 0.001; **** *p* < 0.0001; inf: inferior allele; sup: superior allele.

**Figure 6:**
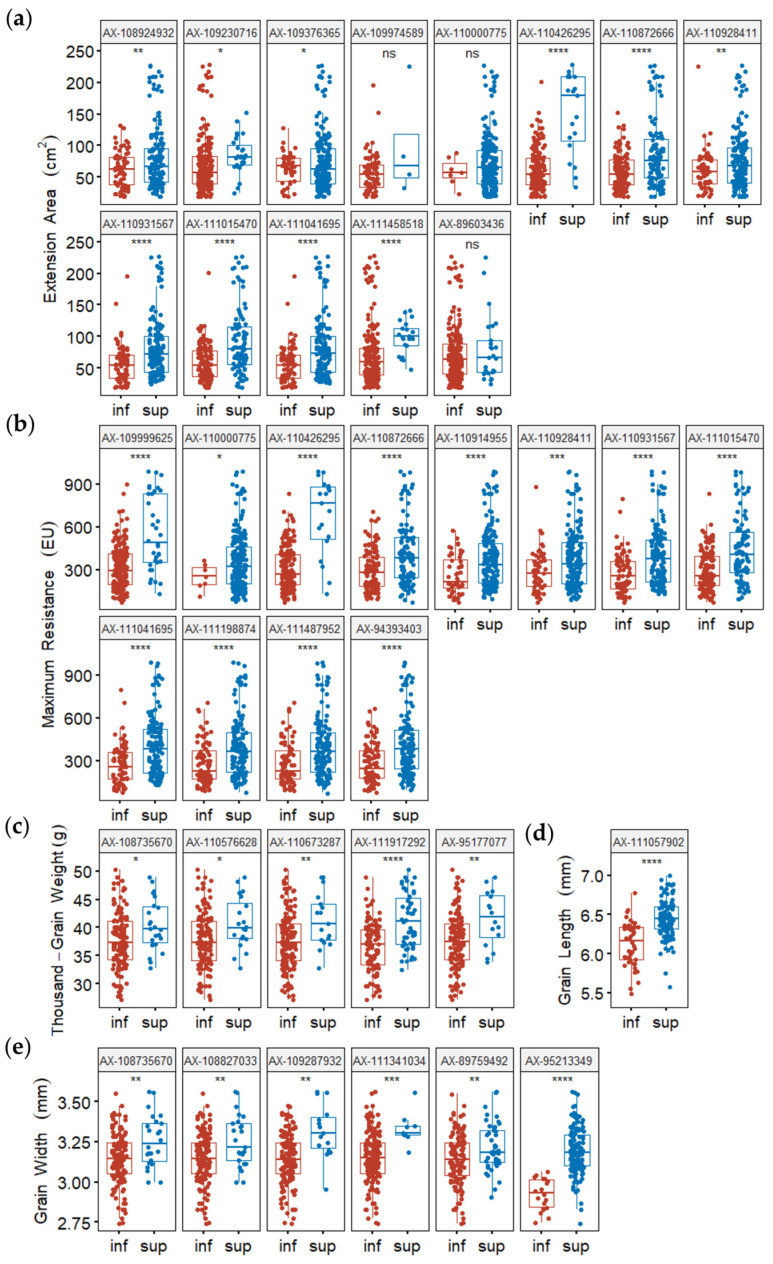
Average phenotypic values of accessions with different alleles for extension area (**a**), maximum resistance (**b**), thousand-grain weight (**c**), grain length (**d**), and grain width (**e**). *p*-values were determined by two-tailed Student’s *t*-tests. ns: no significant difference, *p* > 0.05; * *p* < 0.05; ** *p* < 0.01; *** *p* < 0.001; **** *p* < 00001; inf: inferior allele; sup: superior allele.

**Figure 7:**
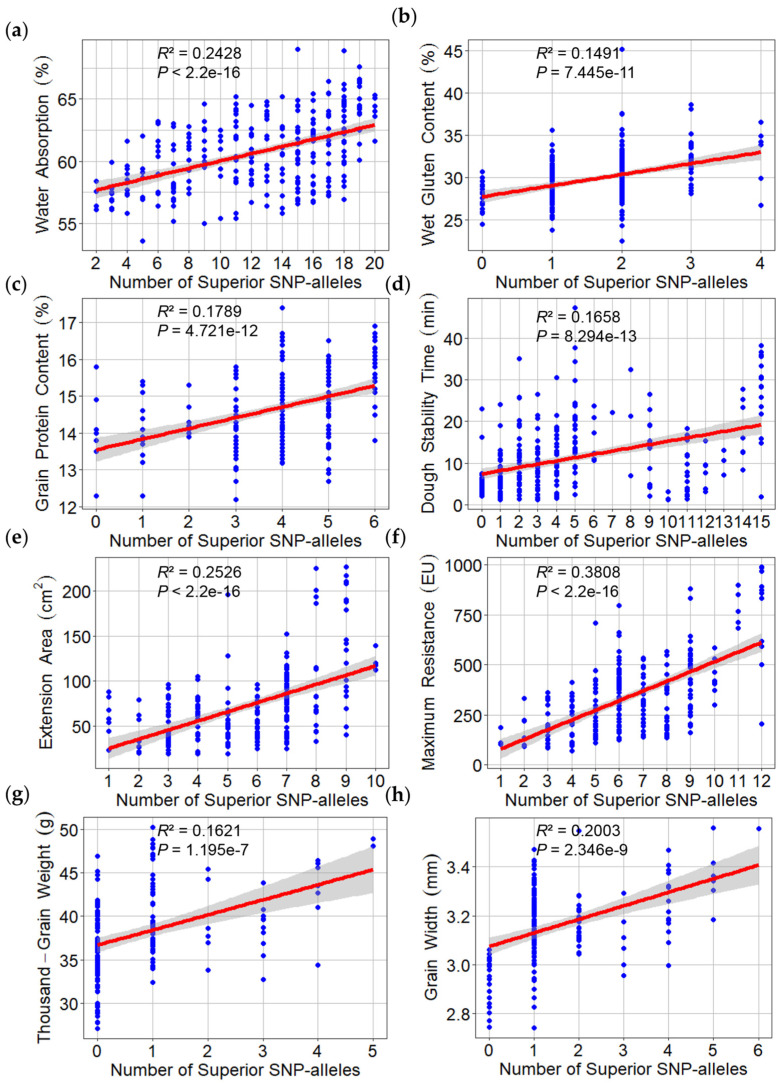
Superior allelic effects of the highest –log_10_ (*p*) single-nucleotide polymorphisms (SNPs) within the quantitative trait loci (QTL) detected by the genome-wide association study (GWAS) based on linear regression analysis for water absorption (**a**), wet gluten content (**b**), grain protein content (**c**), dough stability time (**d**), extension area (**e**), maximum resistance (**f**), thousand-grain weight (**g**), and grain width (**h**). Values shown at the bottom of the box plot correspond to each SNP allele.

To simplify the description of allelic effects, marker alleles associated with a high phenotypic value were defined as “superior alleles”; all other marker alleles were considered “inferior alleles”. To confirm those effects, MTAs with the minimum *p*-value for detected QTL were used to determine whether differences in phenotypic values (grouped according to polymorphism) reached statistical significance. For the most representative MTAs, the phenotypic values of superior alleles were significantly higher than the phenotypic values of inferior alleles, as determined by *t*-tests (*p* < 0.05) (Figure 5 and Figure 6). MTAs representative of detected QTL were used to calculate the number of superior alleles in each wheat line. The patterns of relationships between the number of superior alleles and the phenotypic value were similar for all detected traits, such that the phenotypic value increased with the number of superior alleles: WA (*R*^2^ = 0.2428), WGC (*R*^2^ = 0.1491), GPC (*R*^2^ = 0.1789), DST (*R*^2^ = 0.1658), EA (*R*^2^ = 0.2526), MR (*R*^2^ = 0.3808), TGW (*R*^2^ = 0.1621), and GW (*R*^2^ = 0.2003) (Figure 7).

## 3. Discussion

### 3.1. Identification of MTAs and Comparisons with Previous Findings

In this study, 188 MTAs were identified, representing 87 QTL for six quality-related and three grain yield-related traits. A comparison of these loci with previously identified loci showed that some were in the same region, indicating that our results were reliable.

Among the characteristics of bread, WA plays an important role. A high WA can increase the bread output per unit of flour, increase bread softness, and extend the shelf life of fresh bread. In this study, 21 QTL for WA were detected on chromosomes 1B, 1D, 2A, 2D, 3A, 3B, 3D, 5B, 5D, 6B, 6D, and 7B. *qWA-2A*, represented by *AX-111129364* at 735.35 Mb on chromosome 2A, was located at a position similar to the location of *QLWA.cau-2A*, associated with WA under late sowing time conditions [11]. 

For WGC, four QTL were identified on chromosomes 1B, 3D, 4B, and 5D. *qWGC-3D* included the MTA *AX-110928411* located next to *TaNAC019-D*, which encodes an endosperm-specific NAC transcription factor that regulates the expression of *HMW-GS* (high molecular weight glutenin subunit) and *TaSPA* (storage protein accumulation) genes [25]. The position of *qWGC-3D* was similar to the positions of *qEA-3D.1* and *qMR-3D.1*, suggesting an important role for this locus in regulating wheat quality.

Eight QTL for GPC were identified on chromosomes 4B, 5B, 5D, 6A, and 6B. The position of *qGPC-5B.3* was close to the position of the previously detected QTL *QGpc.caas-5B.1* [26].

For DST, *qDST-1A.3*, represented by seven MTAs, contained the *Glu-1Ax* and *Glu-1Ay* genes that encode HMW-GS [27,28]. The positions of *qDST-4B* and *qDST-5A* were similar to the positions of *QST.caas.4BS* [22] and *QLDST.cau-5A.1* [11], which have been associated with DST.

Most QTL for EA were colocalized with MR-associated QTL, a finding supported by the strong correlation between these two traits. Because few studies have focused on the genetic dissection of flour EA and MR, our identification of the QTL for these traits provides an important reference for studies of flour quality traits. 

Comparative analysis revealed that *qTGW-5A*, a quantitative trait locus for TGW, was mapped to the chromosome 5AL and therefore located near a previously reported quantitative trait locus for GW [29]. Many QTL for TGW have been detected on chromosome 5B [30,31,32,33,34,35]; some of these QTL coincide with QTL for GL and/or GW [30,32,33,34,35]. In the present study, the QTL *qTGW-5B* and *qGW-5B* were detected in a similar region; they were adjacent to *QTkw-5B.1*, a quantitative trait locus for TGW detected in three recombinant inbred line populations [30], and adjacent to a quantitative trait locus affecting TGW, GL, and GW in the Rye Selection 111/ Chinese Spring recombinant inbred population [35]. The TGW QTL *qTGW-5D.1* and the WA QTL *qWA-5D* for WA were present at similar intervals on chromosome 5D, in a region containing *TaARF25-5D*. Notably, *TaARF25-5D* encodes an auxin response factor (ARF) that interacts with *TaIAA21* to regulate wheat grain size and weight development [36]. A comparison of *qGW-2D*, a quantitative trait locus for GW detected in the present study, with previously identified QTL controlling TGW, GL, and/or GW showed that these significant QTL were mapped within an approximately equivalent or contiguous chromosomal region [32,37]. 

To our knowledge, the present study identified several novel QTL: two for TGW (*qTGW-1A* and *qTGW-5D.2*), one for GL (*qGL-4B*), four for GW (*qGW-1A*, *qGW-5A*, *qGW-5D*, and *qGW-7D*), and nearly all wheat quality-related QTL detected in this study (except *qWA-2A*, *qWGC-3D*, *qEA-3D.1*, *qMR-3D.1*, *qGPC-5B.3*, *qDST-1A.3*, *qDST-4B*, and *qDST-5A*).

### 3.2. The Validation of Rare and Multiple Phenotype-Related MTAs in the Association Population

In the present study, 188 MTAs were identified, representing 87 QTL for wheat quality and yield. MTAs with the minimum *p*-value for the detected QTL were used to calculate the frequencies of rare loci. MTAs associated with a high WA, WGC, GPC, EA, and MR, as well as a long DST, were defined as superior alleles; all others were regarded as inferior alleles. The frequencies of 18 superior alleles among the accessions were <15%, indicating that they have not been fully utilized but should receive greater consideration in molecular breeding. For WA, the frequencies of superior alleles of the SNPs *AX-94697975* on chromosome 5B and *AX-109821074* on chromosome 6D among wheat accessions were 12.63% and 8.07%, respectively. For WGC, two rare superior alleles were identified involving the SNPs *AX-111337684* on chromosome 3D (14.04%) and *AX-110581813* on chromosome 5D (8.42%). Among the surveyed accessions, the frequency of the superior allele of the SNP *AX-110469098* for GPC on chromosome 6A was 0.70%, suggesting that this SNP is a very rare locus; it should be more extensively explored and more fully utilized in wheat breeding. Two superior alleles for DST (*AX-109819152* on chromosome 4B and *AX-110978247* on chromosome 5D) and another two for MR (*AX-110426295* on chromosome 2D and *AX-109999625* on chromosome 3D) were present in < 13% of the surveyed accessions. For EA, five superior alleles (*AX-110426295*, *AX-111458518*, *AX-109230716*, *AX-89603436*, and *AX-109974589*) were detected in <11% of the surveyed accessions, although these loci are likely significant for improvements in EA. For example, *AX-110426295* was significantly associated with EA and MR. For TGW, three rare superior alleles (*AX-110673287* on chromosome 1A, *AX-95177077* on chromosome 5A, and *AX-110576628* on chromosome 5B) were present in <15% of the surveyed accessions. For GW, the frequencies of superior alleles of the SNPs *AX-111341034* on chromosome 2D and *AX-109287932* on chromosome 7D among the surveyed accessions were 6.67% and 13.33%, respectively.

Among the detected traits, relatively strong correlations were determined for some phenotypes, whereas 25 MTAs showed pleiotropic effects on multiple phenotypes. For example, WGC shared the MTA *AX-109488694* with GPC on chromosome 4B. DST had one and three SNPs in common with MR and EA, respectively; the SNP *AX-111015470* was associated with both MR and EA. Among the 14 SNPs shared between EA and MR, one was associated with DST, EA, and MR. This result was closely related to the traits of EA and MR, with positive correlations of up to 0.98. Similarly, six MTAs were associated with both TGW and GW; there was a significantly positive correlation between the latter two traits. TGW shared two SNPs on chromosome 5D (*AX-111917292* and *AX-109124553*) with WA, indicating that these SNPs simultaneously control wheat quality and yield. The specific association of some MTAs with several traits suggests that these loci control multiple phenotypes, whereas other loci play key roles in specific phenotypes. The collaborative expression of these QTL can synergistically improve wheat quality and yield.

## 4. Materials and Methods

### 4.1. Materials

This study examined 285 regional test lines of wheat from the year 2020 in Hebei Province, China, the primary province involved in producing strong gluten-containing and high-quality wheat in China.

### 4.2. Measurements of Flour Quality and Grain Traits

A scaled camera-assisted phenotyping system (SC-G, Wanshen Detection Technology Co., Ltd., Hangzhou, China) was used to record grain yield-related traits (TGW, GL, and GW). GPC was measured on 0.2000 g of wholemeal samples using a full-automatic Kjeldahl analysis device (FOSS Kjeltec 8400, Copenhagen, Denmark) according to Chinese standard GB 5009.5-2016. EA and MR were determined using an extensograph (Brabender Extensograph^®^-E, Duisburg, Germany) according to Chinese standard GB/T 14615-2019. When measuring EA and MR, the amount of flour sample used was calculated using the following formula: m = 300 g × 0.86/(100% − MCF), where m was the weight of flour sample; 0.86 was the dry matter content; and MCF was the moisture content of flour. DST and WA were determined using a full-automatic farinograph (Brabender 810151.001, Germany) according to Chinese standard GB/T 14614-2019. WGC was determined on 10.00 g of flour using a gluten measuring system (Perten GM2200, Hägersten, Sweden) according to Chinese standard GB/T 5506.2-2008. During assessments of quality traits, each sample was measured at least twice, and the mean value was used in subsequent analyses.

### 4.3. Phenotypic Data Analysis

All statistical analyses were conducted using R v4.3.3 software. Two groups were compared using two-tailed Student’s *t*-tests. Correlations among traits were calculated using Pearson correlation coefficients.

### 4.4. SNP Genotyping

The fresh leaves of 285 regional test lines of wheat in this study were used to extract DNA with the modified CTAB method [38] for sequencing with the wheat iSelect 15K SNP array http://www.cgmb.com.cn/index.php/Home/Index/ke_suc_case_xiangqing?id=47 (accessed on 1 April 2020) from China Golden Maker Biotechnology Co., Ltd. (Beijing, China) http://www.cgmb.com.cn/ (accessed on 1 April 2020) [13]. This wheat iSelect 15K SNP array contains 13,947 SNPs, of which 1272 are functional markers and 12,674 are genomic coverage markers. For quality control assessments of SNP data, SNPs with a missing rate > 20% and minor allele frequency (MAF) < 5% were deleted using PLINK v1.9 software (https://www.cog-genomics.org/plink2/) (accessed on 1 January 2024) [39]. After filtering, 9138 SNPs remained available for the GWAS. These filtered SNPs were distributed on all wheat chromosomes, with 3211, 3838, and 2089 SNPs in the A, B, and D sub-genomes, respectively. SNP positions were determined via BLAST search IWGSC RefSeq v1.0 2018; http://wheat-urgi.versailles.inra.fr/Tools/BLAST (accessed on 1 January 2023).

### 4.5. Linkage Disequilibrium

LD was calculated as the squared allele frequency correlation (*r*^2^), using PLINK v1.9 software. The LD decay distance and half decay distance were generated with *r*^2^ values for the distances of SNP pairs, using the ggplot2 package in R software.

### 4.6. Population Structure and Kinship Analysis

Three clustering methods were used to assess genetic structures at the population level. First, the population structure was analyzed using the Structure v2.3.4 software, as previously described [40,41]. Second, a phylogenetic tree was generated using TASSEL v5.2.73 software and the neighbor-joining method [42]. Third, PCA was conducted using PLINK v1.9 [39]. The relative kinship matrix was estimated in TASSEL 5.2.73 software using the four-scaled identity-by-state (IBS) method, with a reasonable estimate of additive genetic variance followed by selection using the pheatmap package in R software [42].

### 4.7. Genome-Wide Association Mapping

The GWAS methodology was implemented using the GAPIT package in R software, combined with a mixed linear model (PCA + relative kinship) [43], which controlled for false positives caused by the population structure and relative kinship. The first five principal components were regarded as covariate variables and included in the GWAS model. Relative kinship was determined using TASSEL 5 software [42]. To decrease type II errors, significant MTAs were set to −log10 (*p*-value) ≥ 3.0, as shown in the Manhattan and Q-Q plots [13,44].

## 5. Conclusions

In this study, QTL for quality-related and yield-related traits in wheat were identified through a GWAS of six quality-related traits (WA, GPC, WGC, EA, MR, and DST) and three grain yield–related traits (TGW, GL, GW) in 285 regional test lines of wheat from Hebei Province, China, using the wheat 15 K array. Among the 87 QTL harboring 188 detected MTAs, twenty-five MTAs on chromosomes 1A, 2D, 3D, 4B, 5B, 5D, and 6A were pleiotropic for multiple traits. *AX-111015470* on chromosome 1A exhibited pleiotropic effects on DST, EA, and MR. SNPs *AX-111917292* and *AX-109124553* on chromosome 5D showed pleiotropic effects on wheat WA and TGW. This study also identified several new QTL. In the surveyed accessions, 18 rare superior alleles were identified on chromosomes 1A, 2D, 3A, 3D, 4B, 4D, 5A, 5B, 5D, 6A, 6D, and 7D; these alleles merit greater consideration in wheat breeding. The results of this study provide important genetic information for cultivating high-yield and high-quality varieties of wheat, which will contribute to the development of new wheat varieties with desirable alleles that synergistically improve wheat quality and yield.

## Figures and Tables

**Table 1:** Statistics for water absorption (WA), wet gluten content (WGC), grain protein content (GPC), dough stability time (DST), extension area (EA), maximum resistance (MR), thousand-grain weight (TGW), grain length (GL), and grain width (GW).

Trait	WA (%)	WGC (%)	GPC (%)	DST (min)	EA (cm^2^)	MR (EU)	TGW (g)	GL (mm)	GW (mm)
Mean	60.68	29.99	14.68	11.04	72.34	351.10	38.08	6.35	3.16
Min	53.60	22.50	12.20	1.20	19.00	72.00	27.11	5.49	2.74
Max	69.00	45.20	17.40	47.40	227.00	989.00	50.23	7.00	3.56
SD ^a^	2.93	2.77	0.99	8.62	43.85	195.18	5.04	0.29	0.16
CV ^b^ (%)	4.82	9.23	6.78	78.05	60.62	55.59	13.25	4.59	5.13

^a^ SD, standard deviation; ^b^ CV, coefficient of variation.

## Data Availability

The data and results are available to every reader upon reasonable request.

## Supplementary Materials

The following supporting information can be downloaded at https://www.mdpi.com/article/10.3390/plants13182545/s1, Table S1: the QTL and significant SNPs identified by GWAS for valued traits; Table S2: the QTL and significant SNPs identified by GWAS for grain yield-related traits.

## Abbreviations

QTL	Quantitative trait loci
GWAS	Genome-wide association study
WA	Water absorption
WGC	Wet gluten content
GPC	Grain protein content
DST	Dough stability time
EA	Extension area
MR	Maximum resistance
TGW	Thousand-grain weight
GL	Grain length
GW	Grain width
SNP	Significant single-nucleotide polymorphism
MTA	Marker–trait association
